# The NextGen Study: patient motivation for participation in genome sequencing for carrier status

**DOI:** 10.1002/mgg3.306

**Published:** 2017-07-02

**Authors:** Tia L. Kauffman, Stephanie A. Irving, Michael C. Leo, Marian J. Gilmore, Patricia Himes, Carmit K. McMullen, Elissa Morris, Jennifer Schneider, Benjamin S. Wilfond, Katrina A. B. Goddard

**Affiliations:** ^1^ Center for Health Research Kaiser Permanente Northwest Portland Oregon; ^2^ Department of Medical Genetics Kaiser Permanente Northwest Portland Oregon; ^3^ Department of Pediatrics Treuman Katz Center for Pediatric Bioethics Seattle Children's Hospital and Research Institute University of Washington School of Medicine Seattle Washington

**Keywords:** Decision‐making, genetic carrier testing, genetics, genome sequencing, preconception counseling, survey

## Abstract

**Background:**

While translational genomic sequencing research is increasing, few studies have been limited to healthy individuals; most have focused on patients with a disease or a strong family history of a disorder. The limited studies that have included healthy individuals have focused on the disclosure of medically actionable secondary results, rather than carrier status, to assess reproductive risks. To address this important gap, we conducted the NextGen study, which focuses on carrier status and medically actionable secondary findings in a population of women planning a pregnancy.

**Methods:**

We assessed 310 participants’ motivations for receiving genome sequencing for expanded carrier screening and experiences with familial genetic conditions that may relate to study participation.

**Results:**

Most participants reported that obtaining general health information from genome sequencing was their primary motivator, even though they were recruited to join a study to learn more about carrier status. Forty‐two percent of enrolled women became pregnant prior to obtaining sequencing results.

**Conclusion:**

Genomic carrier testing may need to be offered to women prior to active pregnancy efforts to be useful for reproductive planning.

## Introduction

The majority of genetic testing related to reproduction occurs prenatally through screening tests such as blood tests and ultrasounds and diagnostic tests such as amniocentesis or chorionic villus sampling (American College of Obstetricians and Gynecologists' Committee on Practice Bulletins, 2016). Prenatal screening is an important part of pregnancy management to identify chromosomal abnormalities, while carrier screening plays an important role in identifying risks for autosomal recessive and X‐linked disorders. Waiting until there is a pregnancy for carrier screening results in a missed opportunity to identify and provide an array of options prior to conception.

Historically, carrier testing in clinical care has been limited to ethnically based testing, and professional recommendations for general population screening are limited to cystic fibrosis and spinal muscular atrophy (American College of Obstetricians and Gynecologists' Committee on Genetics, 2017). In recent years, access to expanded carrier screening panels has increased and several commercial companies now offer carrier screening for 100–300 conditions (GoodStart Genetics, 2016; Sequenom, 2016a; Counsyl). These panels are sometimes limited to common known variants in the genes interrogated (Sequenom, 2016b). Despite this rapid increase in availability, these expanded panels are not routinely offered and little is known about the uptake and motivations for broader genomic sequencing for carrier screening.

Translational genomic sequencing research has mostly focused on patients with a disease or a strong family history of a disorder, such as adult or pediatric cancer, childhood developmental disabilities, or cardiac conditions (Biesecker et al. 2009; Foreman et al. 2013; Yang et al. 2013; Gallego et al. 2014; Gray et al. 2016; Green et al. 2016). These studies tend to primarily enroll non‐Hispanic white patients with a high education level and annual income, both as a function of geography and because these populations tend to be early adopters of research and new technology (Facio et al. 2013; Gray et al. 2016; Lupo et al. 2016).

The few sequencing studies that have included healthy participants have focused on the disclosure of medically actionable secondary findings. In the Clinical Sequencing Exploratory Research (CSER) consortium, for example, most studies disclosed carrier status as a secondary (not primary) finding (Green et al. 2016). Our study, called NextGen, on the other hand, focuses on carrier status as the primary indication for sequencing and medically actionable conditions as secondary findings in a population of women and couples planning a pregnancy. This has allowed us to assess our participants' motivations for receiving genome sequencing to determine their carrier status. Participants in one CSER genome sequencing study, ClinSeq, reported two main motivations for joining: general interest in scientific research and a desire to learn about their personal health (Facio et al. 2011, 2013). Studies such as NextGen are important to provide valuable information on whether a healthy preconception population will be motivated to receive genome sequencing and will illuminate the reasons for their interest.

As we began NextGen we hypothesized that women and their partners who had already received clinical carrier screening – usually for cystic fibrosis – would be interested in receiving information about many more autosomal recessive, X‐linked, and mitochondrial conditions to assist them in their planning for a future pregnancy since they had already expressed an interest in carrier screening. In addition to offering results for over 700 carrier status gene/condition pairs(Himes et al. 2017), we also offered disclosure of sequencing results related to medically actionable secondary findings, as recommended by the American College of Medical Genetics and Genomics (Green et al. 2013). We hypothesized that the receipt of secondary findings would be a secondary motivation for joining the study because we presented our study to potential participants as an opportunity to use genome sequencing to learn more about their carrier status for recessive conditions than traditional clinical care screening currently offers. This paper offers insight into the motivations of the women in our healthy preconception population for participation in genome sequencing research.

## Materials and Methods

### Ethical compliance

The study procedures were reviewed and approved by the Kaiser Permanente Northwest (KPNW) Institutional Review Board. All participants provided written consent for participation and received written information on study procedures. This research was conducted as part of the National Human Genome Research Institute (NHGRI) CSER consortium.

### Study overview

The detailed study design has been published elsewhere (Kauffman et al. 2017). Briefly, we identified women at KPNW in the Portland, Oregon metropolitan area who were not pregnant, but planning a pregnancy and had carrier screening completed as part of either a preconception visit or who had the test during a prior pregnancy and were at least 6 months postpartum. Women were called and asked if they were interested in joining a study to receive carrier screening for approximately 700 conditions and approximately 150 medically actionable secondary findings. Interested women were mailed the study consent form and a consent visit was scheduled. If a woman consented to join the study during an in‐person visit with a genetic counselor that lasted about 30 min, she then completed a survey and was randomly assigned into a study arm: genome sequencing (GS) or usual care (UC), which was the clinical genetic test they had already received and routine clinical care.

Because the data presented in these analyses were collected prior to randomization, women from both study arms were included. While couples represented a portion of the NextGen study population, we did not include the male participants in this analysis. Due to differences in recruitment methods by sex, the male participants in NextGen may not reflect the motivations of general healthy adult males for receiving carrier screening via genome sequencing.

### Data collection

The potential participants met with one of three study genetic counselors for an approximately 30 minute informed consent and education session. These genetic counselors were board certified practitioners in the KPNW system with 4–27 years of experience. During the meeting, the genetic counselor explained basic genetic inheritance patterns, discussed the benefits and limitations of genome sequencing, reviewed the consent form, and answered any participant questions. Toward the end of the meeting, following the educational discussion and consent form review but prior to randomization, all potential participants who signed the consent form were asked by the genetic counselor two open‐ended questions regarding their motivation for study participation: “What are you hoping to learn from being in this study?” and “Are there specific conditions you are hoping to learn about through genome sequencing? Please describe.” We chose to have the genetic counselor ask these two questions rather than embed them in a questionnaire to facilitate further clarification and discussion about the study, such as correcting misunderstandings about the scope of results that would be returned, and to minimize nonresponses. The genetic counselors documented responses to these open‐ended questions on a study form, and data were subsequently entered in Microsoft Excel. Responses were not always recorded verbatim, but were documented in sufficient detail similar to how genetic counselors document traditional patient encounters in the electronic medical record. All quotes shared in the manuscript represent responses as they were documented by the genetic counselor.

A survey was administered online toward the end of the meeting, prior to randomization, which included two questions related to experiences with genetic conditions: “Do you know a family of a child with a genetic condition?” and “Are there any genetic conditions in your family?” For any responses of yes to the latter question, participants were subsequently asked the open‐ended question “What is/are the condition(s)?”

### Analysis

We conducted a content analysis of the recorded responses to the open‐ended questions to identify key themes (Silverman 2009; Bernard and Ryan 2010; Denzin and Lincoln 2011). An initial reading of the responses was conducted by author SI, followed by a second reading by authors SI, MJG, and PH to establish a draft list of codes (e.g., descriptive phrases that summarized the content). The draft list of codes was discussed with the project team until consensus was met on codes and their related definitions. Authors MJG, PH, TLK, and SI individually re‐read and applied the codes to the responses, with at least two people reviewing each response. All discrepancies were reconciled to 100% agreement. The responses could reflect a single or multiple codes. The codes were summarized by SI into themes representing motivations for joining the study, which were then tabulated by frequency. The themes were shared with the project team for comment and consensus. When responses to the question about specific conditions included secondary findings (related to personal health exclusively), we coded the specific conditions into broad disease categories using the same process.

We calculated frequencies of responses to the survey questions. We analyzed participant demographics for all participants. We calculated the means, standard deviations, and Pearson correlations for continuous variables.

## Results

The analyses included 310 women. The participants were mostly white, non‐Hispanic, highly educated, and employed. Eighty percent were married and 45% had children. The average age of enrolled women was 32.1 years (range 21–46 years); 95.5% of participants were <40 years old. Compared to the general population of women within KPNW who became pregnant during the recruitment period (*n* = 16,742), women who enrolled in the study were older, more likely to be white, and more likely to have higher education and higher income (Table 1). On average, results were disclosed to a participant 103 days after consent (range 41–187 days). Between consent and sequencing result disclosure to both women and their male partners, 42% of the women became pregnant.

**Table 1:** NextGen participant demographics (*N*, column %)

	Study females, *N* = 310	KPNW pregnant females^a^, *N* = 16,742
Mean age, years (SD)	32.1 (4.4)	28.9 (6.0)
Race		
White	242 (78)	11,949 (71)
Non‐white/Multiple	63 (20)	4793 (29)
Ethnicity		
Hispanic/Latino	23 (7)	1939 (12)
Not Hispanic/Latino	282 (91)	14,803 (88)
Education		
Less than bachelor's degree	76 (25)	11,786 (70)^b^
Bachelor's degree	114 (37)	3239 (19)
Graduate degree (MS, JD, MD, PhD)	120 (39)	1717 (10)
Employment		
Employed	265 (85)	N/A
Unemployed	8 (2)	N/A
Other (homemaker, student, retired)	34 (11)	N/A
Marital status		
Currently married	246 (80)	N/A
Never married	46 (15)	N/A
Other (widowed, divorced, separated)	12 (4)	N/A
Children		
Currently has children (% yes)	138 (45)	N/A
Income		
Less than $80,000	109 (35)	9738 (58)^c^
$80,000–$149,999	143 (46)	5420 (32)
$150,000 or more	42 (14)	1583 (9)

Category totals do not equal 100% due to exclusion of missing responses; N/A = not available.

a Women in the KPNW patient population with pregnancies initiated during the study recruitment period (Feb 1, 2013 to Oct 31, 2015).

b Using United States census geocoded data.

c Income less than $75,000 using United States census geocoded data.

In the self‐administered survey, participants described their experience with “genetic conditions” in their family and families of people they know. Twenty‐three percent reported genetic conditions within their immediate or extended family. However, most of the examples provided by participants were unlikely to be monogenic conditions (e.g., diabetes, heart disease). While 49% reported knowing a family with a child with a participant‐defined genetic condition, it is unknown if the conditions were actually monogenic disorders.

### “What are you hoping to learn?”

The 292 participant responses to the first open‐ended question were categorized into three key themes: general information, reproductive planning, and support of research (Table 2). Of these participants, 118 (40%) had responses that fell into more than one theme. The most common reason for study participation was general information (215/69%). For example, participants stated that it is “beneficial to have more information” or that they were “very curious” and may not have provided specific concerns. Although few women mentioned specific conditions in response to this question, those that did were categorized under general information. The second most common reason identified pertained to reproductive planning (160/52%). Most participants provided statements on the utility of the results for family planning (e.g., “Knowledge is helpful for family planning” or “How genetics would impact reproductive planning”). Some of these respondents specifically mentioned risks to future children or pregnancies (e.g., “Interested in risks for a child with problems” and “potential problems in a fetus”). These categories (general information and reproductive planning) contained a fair amount of overlap. For example, 43% of those stating general information reasons also reported reproductive planning reasons for participation, and made comments such as, “I am curious about genetic testing. Perhaps the results will be helpful for family planning.” Conversely, 60% of those mentioning reproductive planning also mentioned that general information was a motivation for participation. Support of research was the third most commonly stated motivation for participation (34/11%) and included such comments as, “Want to help advance science” and “Medical research is important.” Several participants mentioned backgrounds or careers in science as part of their motivation for participation (e.g., “Want to help with research in general, since I have a science degree”).

**Table 2:** NextGen participant motivation: “What are you hoping to learn from being in this study?”^a^

Theme	Definition	*N* = 310	Example(s)
General information	General health information seeking	215 (69%)	General curiosity; The more information the better; Son has autism, wants information
Reproductive planning	Information seeking related specifically to pregnancy or reproductive planning, or investigation of fertility	160 (52%)	Information for planning for a family; Reproductive risks; Risks for child with problems; Interested in genetics after multiple spontaneous abortions
Support of research	General support of scientific research or advancement of technology	34 (11%)	On board with helping science; Helping the greater good is important; Contributing to research
Unknown	No response or information provided	18 (6%)	Nothing in particular

a Responses not mutually exclusive.

### “Are there specific conditions you hope to learn about?”

For the open‐ended question about specific conditions, we coded responses into the following five categories: carrier status (e.g., “Fragile X”), secondary findings (e.g. “autoimmune conditions for her”), unknown family history (e.g., “Doesn't know dad's family history”), conditions related to known family history of disorder (e.g., “Mother has hemochromatosis”), or no response (Table 3). Of the 184 participants who reported that there was a specific condition they were hoping to learn about, 102 (55%) of participants’ responses were coded in more than one category (e.g., “Nieces with galactosemia” was coded both in carrier status and conditions related to known family history of a disorder). The most common combination was “secondary findings” and “conditions related to known family history of disease” with comments such as “Breast cancer in family” and “Curious if she has ‘colon cancer’ gene since she has two relatives with colon cancer”; this combination was recorded for 84/102 participants with multiple responses (82%).

**Table 3:** NextGen participant motivation: “Are there specific conditions you are hoping to learn about through genome sequencing?”^a^

Theme	Definition	*N* = 310	Example(s)
Secondary findings	Condition mentioned is related to participant's personal health	140 (45%)	Anything that might impact my health; Specific conditions – see Fig. 1
Family history: known	Condition known in family members	93 (30%)	Specific conditions listed in various family members: parent, aunt, grandparent, cousin, nephew
Carrier status	Condition mentioned is related to carrier status, including reproductive risk	50 (16%)	Wants to avoid the burden of genetic diagnosis; Specific conditions – cystic fibrosis, thalassemia, muscular dystrophy, etc.
Family history: unknown	Total or partial unknown family history, including adoption or uncertain parentage	9 (3%)	No history on grandfathers; Husband is adopted and no information about family history; Father is adopted
Nothing	No specific concerns or conditions given	126 (41%)	Nothing in particular

a Responses not mutually exclusive.

**Figure 1:**
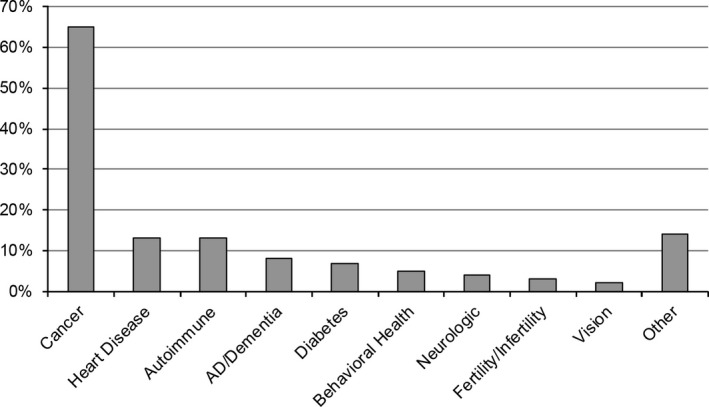
Specific health conditions or categories mentioned among NextGen participants interested in secondary findings from genome sequencing.

Interest in secondary findings was the most common response to this question (140/45%), and participants mentioned a wide variety of conditions related to the participants’ own health. These conditions were coded into disease categories and are displayed in Fig. 1. Cancer (any type) was the most commonly reported disease or condition indicated by respondents with an interest in additional findings (91/140 or 65%). Of the 91 cancer responses, 54 (59%) contained references to traditionally or solely female cancers (breast, ovarian, *BRCA*, “the breast cancer gene”). Of the 54 women who listed female‐specific cancers, 36 (67%) indicated a family history of that cancer. Twenty of 140 participants (14%) responded with a secondary finding that did not fit into one of the predefined disease categories, including poor vision, mental health issues, gallbladder problems, and Ehlers‐Danlos syndrome. Of the 140 participants who reported an interest in secondary findings, 36 (26%) of these responses included more than one disease category (e.g., cancer and heart disease).

Carrier status was mentioned by 50 (16%) of participants. This included specific conditions (e.g., cystic fibrosis) and more general genetic diagnosis concerns (e.g., “wanting to avoid the burden of a genetic diagnosis” or “anything that would impact future children”). Responses captured in all categories included genetic information disclosed through study genome sequencing and information not currently available via sequencing (e.g., causes of infertility and autism).

## Discussion

This study illuminated healthy individuals’ motivation for receiving genome sequencing. Our participants reported strikingly similar motivations for joining compared to other translational genome sequencing studies, a result that is surprising since most other related studies recruited participants with a known disease who were seeking a diagnosis for a suspected genetic disorder based on phenotype (Green et al. 2013). The most common response to motivation for participation in both the CSER ClinSeq study and ours was a desire to obtain general information (Facio et al. 2011). It was surprising that reproductive planning was not the most common motivation for joining NextGen, especially since all women enrolled in the study had already received clinical carrier testing, were recruited into a study to provide expanded carrier testing, and seemingly would be primarily interested in learning about additional carrier results.

In these women actively planning a pregnancy, pending carrier status results do not seem to be associated with reproductive delay, as evidenced by 42% of couples conceiving between study enrollment and their sequencing results disclosure. This supports participants’ responses that carrier results are not their primary motivation for undergoing genome sequencing, because if the results were going to be used for conception planning, results from both the female and male would be needed in order to assess pregnancy risks. This indicates that if the goal of preconception carrier screening is to proactively identify at‐risk couples to allow for increased reproductive decision‐making, offering genome sequencing for carrier status at the time of a preconception planning or infertility visit is too late in the process. These women are actively planning a pregnancy and may not delay reproductive plans while waiting for their results. Additionally, because nearly half of pregnancies are unplanned (Finer and Zolna 2016), offering testing at a preconception planning visit will miss a substantial portion of women of childbearing age. However, future research is needed to see if women are interested in carrier screening earlier in their reproductive years, because personal utility in the absence of actively planning a pregnancy may be low.

When asked if there were specific conditions they hope to learn about, only 16% of participants mentioned an autosomal recessive or X‐linked carrier condition. Instead, women more frequently mentioned a secondary finding, overwhelmingly cancer – either broadly or specific cancer types. This is likely due to several factors: (1) many genetic conditions are very rare, and outside of a handful of more well‐known conditions such as cystic fibrosis (for which all had already received clinical testing prior to enrollment), most people cannot name any autosomal recessive or X‐linked disorder; (2) we primed women to be aware of cancer as a secondary finding during the recruitment and consent process, since Hereditary Breast and Ovarian Cancer (HBOC) mutations are fairly widely known and an easily accessible way to explain medically actionable results to lay participants; (3) in recent years, HBOC has been extensively discussed in the popular media (Staudigl et al. 2016); (4) there could be a misconception that a higher percentage of cancer is inherited than caused by other biological and environmental mechanisms, especially if there is a family member that has had cancer (American Cancer Society, 2015–2016); and (5) people have a disproportionate fear of cancer (Metlife Foundation, 2011; American Institute for Cancer Research, 2016).

Results from the survey question about genetic conditions in their family revealed that many people used very broad definitions of “genetic conditions” and included conditions such as autism and heart disease. Respondent perceptions of these being genetic conditions highlights broad inclusivity, using “genetic conditions that run in their family” to mean any condition that runs in their family, regardless of whether or not it is a monogenic disorder with a potential high penetrance disease‐associated variant that could be identified using genomic carrier screening. Interest in learning about genetic causes for nonautosomal recessive disorders, while outside of the scope of the study, still could have been a motivation for enrollment.

Consistent with other translational genomic research studies, our population is majority white, highly educated, with high annual income (Facio et al. 2013; Gray et al. 2016; Lupo et al. 2016). While our population of preconception women is slightly older than the general pregnant population at KPNW during the same timeframe, our patients are much younger than other translational genomic research studies and represent a healthy young adult population (Biesecker et al. 2009; Lupo et al. 2016). Participants in the CSER MedSeq and ClinSeq studies were almost twice as old (*M *=* *56 years of age in both studies) as our population, and had cardiomyopathies or were comprised of a population enriched for atherosclerotic heart disease (Biesecker et al. 2009; Lupo et al. 2016). The MedSeq study and Mt. Sinai Medical Center HealthSeq study populations both included healthy cohorts, but participants were an average of 55 and 48 years of age, respectively. Carrier testing was likely of less interest to these other research cohorts since the majority of participants were past reproductive age (Lupo et al. 2016; Sanderson et al. 2016). This study adds to our knowledge about the motivations for participation in genome sequencing in a younger, healthy population.

### Limitations

Open‐ended responses from participants were obtained from three different genetic counselors and were not recorded verbatim. However, the genetic counselors are professionally trained and experienced in documenting patient comments in the electronic medical record as part of their role in standard care. Because the full depth of the response may not have been recorded or fully elicited from the participant (e.g., if they responded “breast cancer” it may be unclear if they are interested due to family history, an erroneous assumption that most breast cancer is due to *BRCA* mutations, or a clear grasp of what genome sequencing can tell them about their inherited breast cancer risk), the reasons for participation documented here may not be comprehensive. In these cases, we used the most conservative coding (e.g., “breast cancer” would only be coded as an incidental finding for cancer) to ensure we were not over interpreting responses. While women may have been primed during the recruitment process to mention interest in HBOC, the majority (67%) of women who indicated they were interested in a female cancer as a secondary finding mentioned a specific family history in their response. This makes it unlikely that the recruitment priming was the sole motivator for the over‐representative interest in cancer as a secondary finding. Our participants consented to clinical carrier testing as an eligibility requirement for participation in the study and may not represent the views of those who had declined or had not been offered clinical testing. Our population lacks diversity in terms of race/ethnicity, education level, marital status, and income. Our ability to broadly apply our findings to a more diverse population is limited.

## Conclusion

Women planning a future pregnancy who are interested in genomic carrier screening have motivations for obtaining genomic screening that are very broad and not limited to pregnancy planning. Future research should strive to increase diversity in the populations receiving sequencing to see if their reasons for utilizing the technology differ. Additionally, future genomic carrier screening research should offer screening to women earlier in their childbearing years to allow for result disclosure and subsequent reproductive planning options prior to actively attempting a pregnancy.

## Conflict of Interest

SAI reports previous, unrelated research support from Medimmune; no conflicts exist with the current work. TLK, MCL, MJG, PH, CKM, EM, BJW, and KABG do not have any conflicts to disclose. The study sponsor, National Human Genome Research Institute, approved the study design and encouraged use of measures common to other sites in the Clinical Sequencing Exploratory Research consortium, but did not have input as to the data analysis, interpretation, or writing of this manuscript.
